# Stigma towards a Neglected Tropical Disease: Felt and enacted Stigma Scores among Podoconiosis Patients in Northern Ethiopia

**DOI:** 10.1186/1471-2458-13-1178

**Published:** 2013-12-13

**Authors:** Kebede Deribe, Sara Tomczyk, Elizabeth Mousley, Abreham Tamiru, Gail Davey

**Affiliations:** 1 Brighton and Sussex Medical School, Falmer, Brighton, UK; 2 School of Public Health, Addis Ababa University, Addis Ababa, Ethiopia; 3 Institute of Tropical Medicine, Antwerp, Belgium; 4 International Orthodox Christian Charities, Debre Markos, Ethiopia

**Keywords:** Podoconiosis, Stigma, Discrimination, NTD, Ethiopia

## Abstract

**Background:**

Podoconiosis, or non-filarial elephantiasis, is a neglected tropical disease (NTD) characterised by swelling of the lower legs. When left untreated, this disfiguring condition has a significant social impact. This study aimed to describe the stigma experience among podoconiosis patients in Dembecha, Northern Ethiopia and assess potential associations between stigma and sociodemographic determinants.

**Methods:**

The study was conducted in May 2012 in Northern Ethiopia. A questionnaire-based cross-sectional study design was used and stigma was assessed using a validated podoconiosis stigma scale including 'felt’ and 'enacted’ stigma domains. Enacted stigma includes the experience of discrimination such as abuse, loss of employment or prejudicial attitudes, while felt stigma is the perceived fear of enacted stigma. A multivariable linear regression model was used to explore determinants that may be associated with stigma.

**Results:**

A total of 346 clinically confirmed podoconiosis patients participated in the study. The total mean score of all stigma scale items was 30.7 (Range = 0 to 96). There was a higher mean score of scale items in domains of felt stigma (21.7; Range = 0 to 45) as compared to enacted stigma (9.0; Range = 0 to 51). The total mean score of all stigma scale items appeared to increase with disease stage. A final adjusted linear regression model found an association between stigma and factors including monthly income, duration lived in the current residence, and disease stage, after controlling for confounders.

**Conclusion:**

Podoconiosis is a stigmatized disease with a clear social impact. This paper documented the burden of podoconiosis-related stigma and identified associated factors. Programs aimed at preventing and treating podoconiosis should incorporate interventions to mitigate both felt and enacted stigma. Interventions targeting patients should prioritize those with advanced disease.

## Background

Podoconiosis, or non-filarial elephantiasis, is a neglected tropical disease (NTD) characterised by swelling of the lower legs. In addition, affected individuals can develop disfiguring symptoms such as nodular skin changes, a mossy appearance around the base of the foot or foul-smelling wounds. It often affects barefoot subsistence farmers exposed to irritant volcanic soils [1]. Current evidence also suggests genetic susceptibility [1,2]. Podoconiosis is found across Asia, South America, and tropical Africa including Ethiopia where an estimated one million people are affected. Podoconiosis is a chronic condition, leading to progressive disability. However, it is reversible and preventable with hygiene and the use of shoes [3,4].

When left untreated, this disfiguring condition has a significant social impact. Affected people often face forms of severe stigma such as isolation, exclusion from community events and barriers to employment, education, or marriage [5,6]. Podoconiosis is poorly understood by the community and health professionals, and various misconceptions exist about its aetiology, transmissibility, and treatment [7]. In northern Ethiopia, 13% of podoconiosis patients experienced one or more forms of social stigma [8]. In Southern Ethiopia, more than 50% of community members interviewed in one study showed stigmatising attitudes towards podoconiosis-affected individuals while 100% of health professionals interviewed in a second study held one or more stigmatising attitudes [7,9]. Fear of further stigmatization among podoconiosis patients has been found to be a barrier to consistent shoe use (a control intervention) and participation in research such as podoconiosis genetic studies [10,11]. Coping strategies used by patients while facing such stigma include active or avoidant behaviors and efforts to change relational meaning [12]. We have previously reported reduction in quality of life among podoconiosis patients as compared to healthy controls [13].

Stigmatization of podoconiosis patients is a clear example of health-related stigma. Health-related stigma has been described by Weiss *et al*. as “a social process or related personal experience characterized by exclusion, rejection, blame, or devaluation that results from experience or reasonable anticipation of an adverse social judgement about a person or group identified with a particular problem” [14]. This can lead to social, health, and psychological consequences [15]. In particular, stigma-related structures of exclusion can lead to harmful delays in diagnosis or treatment-seeking due to fear, shame, or lack of economic capital (as a result of social exclusion) [16]. Health-related stigma as described above can include enacted and felt stigma; enacted stigma includes the experience of discrimination such as abuse, loss of employment or prejudicial attitudes [17,18], while felt stigma is the perceived fear of enacted stigma [17,19].

Health-related stigma has been described for other major NTDs including lymphatic filariasis (LF), schistosomiasis, onchocerciasis, leishmaniasis, Buruli ulcer and leprosy [20-29]. Studies have described NTD-related stigma experiences in a broad range of domains. Stigma among leprosy patients has been well-studied and provides examples of stigma in domains such as mobility, domestic life, interpersonal interactions and relationships, community, social and civic life and other major life areas [30,31]. For other NTDs, individual studies have focused on particular stigma experiences such as social isolation (as in the case of LF and Buruli ulcer) or issues of self-esteem (as in onchocerciasis) [21,27,32]. Individual symptoms of NTDs have been noted as the source of stigma such as haematuria in urinary schistosomiasis, Buruli ulcer or onchocercal skin lesions, leishmaniasis scars and LF lymphoedema or hydrocele [22,24-26]. As a consequence of this stigma, many studies have reported delayed treatment due to shame and lack of household support or economic means [15,21,27,33].

Potential determinants of stigma related to NTDs have also been explored. Young age and increasing disease stage were found to be risk factors for stigma among various studies. In a study by Yanik *et al*., younger generations affected by cutaneous leishmaniasis showed less acceptance of stigma than older adults [34]. Age and social role appeared to influence social connectedness among LF patients in a study by Person *et al*. [29]. In the same study, stigma appeared to be correlated with increasing disease stage [29]. Higher grades of lymphoedema and hydrocele were also associated with increased stigma in a study by Kumari *et al*. [35]. Other studies found that stigma scores were not associated with any personal characteristics or disease factors as described by Brieger *et al*. in the case of onchocerciasis [32]. Vlassoff *et al*. looked particularly at gender and stigma of onchocercal skin disease across Africa, but found no significant differences [25]. This study aimed to determine the scale of stigma among podoconiosis patients in Dembecha, Northern Ethiopia and assess potential associations between stigma and other factors.

## Methods

The study was conducted in May 2012 in Dembecha woreda (district), Northern Ethiopia. Dembecha is part of West Gojjam zone in Amhara Region State and has a population of 142,118. The woreda consists of 29 kebeles (smallest administrative unit) and the majority of residents come from the Amhara ethnic group (99.82%), use Amharic as their first language (99.87%), and identify themselves as Ethiopian Orthodox Christians (98.47%) [36].

A questionnaire-based cross-sectional study design was used. Stigma was assessed using a validated podoconiosis stigma scale in the domains of felt (15 scale items) and enacted stigma (17 scale items). In previous testing, the scale showed good consistency (i.e. Cronbach’s alpha greater than 0.7) and satisfactory validity with modest correlation between items. Significant correlation existed between felt and enacted stigma patient scales (Spearman’s r = 0.892, p < 0.001) [17].

Podoconiosis treatment services (patient self-help groups, education on foot hygiene, wound care, bandaging, minor surgery and provision of subsidized shoes), were offered for the first time in the study area during 2011 as part of the International Orthodox Christian Charities (IOCC) Gojjam Podoconiosis Project. Prior to these services, a baseline survey was conducted in Dembecha woreda and identified 1,704 cases of podoconiosis from 51,017 screened individuals (i.e. prevalence in individuals greater than 15 years was 3.3%) [6]. Seven kebeles in Dembecha woreda with the highest prevalence estimates from the baseline survey were selected for this study. Patients included in the previous baseline survey were then contacted again for this study. Convenience sampling was used to select the first 50 available patients from each kebele list (i.e. a near-total sample). Random selection was not feasible due to the distribution of patients over considerable distances within each kebele. Individuals under 15 years were excluded. The total study sample included 346 clinically confirmed adult podoconiosis patients.

The questionnaire was piloted before the study using seven patients within the town of Debre Markos. Translation and understanding of the scale was reviewed and corrections were made to ensure clarity. Data collection was conducted for five days from 3^rd^ to 7^th^ May 2012. Seven nurses (one per kebele) were responsible for data collection. These nurses were familiar with the local area, fluent in Amharic, and had sufficient medical background knowledge. They received training on podoconiosis basics, the objective of the study, the potential impacts of the study, details about the podoconiosis scale, patient selection and informed consent. The nurses used the scales for structured interviews because patient low literacy levels prevented self-completion. Additionally, three supervisors, oversaw the data collection process, answered queries and reviewed scale reliability.

SPSS-19 was used for the data analysis. Data were checked, cleaned, and missing scale values were replaced with the items’ average. Data were analyzed using STATA version 11. A descriptive analysis was first conducted to define the basic characteristics of the podoconiosis cases. Stigma was estimated by mean scores in: (1) total stigma scale items, (2) domains of felt stigma only, and (3) domains of enacted stigma only. Stigma was then considered the main outcome and mean scores were calculated for comparison with previous podoconiosis stigma studies conducted in other Ethiopian regions. The Wilcoxon rank sum and Kruskal-Wallis tests were used to analyse mean stigma scale scores according to sociodemographic determinants and other factors. Stigma scores were treated as continuous variables and a multivariable linear regression model was used to explore factors that may be associated with stigma. All variables were included in the model according to conceptual background knowledge and cross-tabulations (ie. age, education, marital status, place of residence, duration lived in current residence, socioeconomic status, and disease stage). All variables remained in the final model with p-values less than 0.05 were considered significantly associated with overall stigma.

Ethical approval was obtained from Amhara Regional Health Bureau in Northern Ethiopia and the Research Governance and Ethics Committee of Brighton and Sussex Medical School. Written informed consent was sought from all participants including thumbprints for those unable to sign. Consent from parents or guardians was obtained for individuals aged <18 years. All data were anonymised and kept on a password-protected area. Identifiable information was stored separately from other research data.

## Results

The descriptive analysis can be seen in Table 1. The age of cases ranged from 15 to 80 years and the mean was 45.7 years (standard deviation [SD] =13.2). There were more female cases (59.8%) than male, and the majority lived in a rural area (98.0%), worked as farmers (72.8%) and were illiterate (76.6%). Approximately half of the cases were married (57.2%) and had no formal schooling (56.8%). All of the cases had an Amhara ethnic background (100%). Household income ranged from 0 to 1500 ETB per month and the mean was 285.14 ETB per month (SD = 206.42).

**Table 1:** Basic characteristics of the podoconiosis patients in Dembecha, Northern Ethiopia, May 2012 (N = 346)

**Variables**		**Number (%)**
Gender	Male	139 (40.2)
	Female	207 (59.8)
Age (years)	Mean *(SD)	45.74 (13.2)
	15–24	10 (2.9)
	25–34	63 (18.2)
	35–44	79 (22.8)
	45–54	92 (26.6)
	55–64	75 (21.7)
	65+	27 (7.8)
Area of Residence	Rural	339 (98.0)
	Urban	7 (2.0)
Occupation	Farmer	252 (72.8)
	Traders	36 (10.4)
	Employed	6 (1.7)
	Daily Labourer	21 (6.1)
	Housewife	0 (0.0)
	Student	4 (1.2)
	Jobless	16 (4.6)
	Other	11 (3.2)
Ethnicity	Amhara	346 (100.0)
Educational status	Not literate	265 (76.6)
	Able to read and write	46 (13.3)
	Primary (grade 1–6)	27 (7.8)
	Secondary (grade 7–12)	8 (2.3)
Household Income (Birr/month)	Median (range)*	296.8 (0–1500)
	1^st^ quintile (poorest)	86 (24.9)
	2^nd^ quintile	77 (22.3)
	3^rd^ quintile	79 (22.8)
	4^th^ quintile	56 (16.2)
	5^th^ quintile (richest)	48 (13.9)
Marital Status	Married	198 (57.2)
	Unmarried	30 (8.7)
	Divorced	63 (18.2)
	Widowed	55 (15.9)
Other Co–morbidities	Yes	64 (18.5)
	No	282 (81.5)
Podoconiosis Disease Stage	Stage 1	51 (14.9)
	Stage 2	175 (51.2)
	Stage 3	93 (26.9)
	Stage 4	15 (4.3)
	Stage 5	8 (2.3)

*Median age = 45 years (interquartile range [IQR] = 35 to 55 years) and median household income = 296.75 ETB per month (IQR = 107.50 to 370.00 ETB).

**Figure 1:**
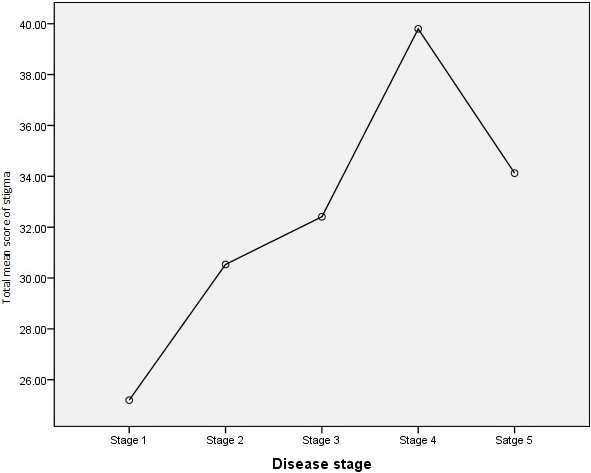
Total mean score of stigma according to podoconiosis disease stage among patients in Dembecha, Northern Ethiopia, May 2012 (N = 346).

The total mean score of all stigma scale items was 30.7 (Range = 0 to 96) as seen in Table 2. There was a higher mean score of scale items in domains of felt stigma (21.7; Range = 0 to 45) as compared to enacted stigma (9.0; Range = 0 to 51). In the felt stigma domains, the highest mean score was found in scale items related to interpersonal interactions (17.6; Range = 0 to 21). In the enacted stigma domains, the highest mean score was found in scale items related to community, social, and civic life (3.9; Range = 0 to 18). The felt and enacted stigma scales correlated significantly (Spearman’s r = 0.626; p < 0.001). As seen in Figure 1, the total mean score of all stigma scale items appeared to increase with disease stage, peaking at stage 4 and decreasing at stage 5.

**Table 2:** Mean stigma scale scores from podoconiosis patients in Dembecha, Northern Ethiopia, May 2012 (N = 346)

**Category**	**Mean (SD)**	**95% CI**
Total stigma scale items		
Total: Mean score of all stigma scale items ranging from 0 to 96	30.7 (0.1)	29.3–32.2
Felt stigma: Mean score of scale items ranging from 0 to 45	21.7 (0.3)	21.2–22.3
Enacted stigma: Mean score of scale items ranging from 0 to 51	9.0 (0.5)	8.0–10.0
Domains of felt stigma only		
Interpersonal interactions: Mean score of scale items ranging from 0 to 21	17.6 (0.1)	17.3–17.9
Major life areas: Mean score of scale items ranging from 0 to 15	2.4 (0.2)	2.0–2.8
Community, social, civic life: Mean score of scale items ranging from 0 to 9	1.8 (0 .1)	1.5–2.0
Domains of enacted stigma only		
Interpersonal interactions: Mean score of scale items ranging from 0 to 11	2.2 (0.1)	1.9–2.5
Major life areas: Mean score of scale items ranging from 0 to 18	2.8 (0.2)	2.4–3.3
Community, social, civic life: Mean score of scale items ranging from 0 to 18	3.9 (0.2)	3.6–4.3

Mean stigma scores according to sociodemographic determinants and other factors are summarised in Table 3: Those living in an urban area had a statistically significant higher stigma score across total stigma scale items, in domains of felt stigma, and in domains of enacted stigma than those living in a rural area (p = 0.013, p = 0.006, p = 0.036, respectively). A statistically significant difference was also found across disease stages in total stigma scale items, in domains of felt stigma, and in domains of enacted stigma (p = 0.005, p = 0.002, p = 0.001, respectively) and across household income groups in domains of enacted stigma only (p = 0.001).

**Table 3:** Mean stigma scale scores according to sociodemographic determinants and other factors among podoconiosis patients in Northern Ethiopia, May 2012 (N = 346)

**Variable**		**Mean stigma scores**					
		**Total stigma**	P-value*	**Felt stigma**	P-value*	**Enacted stigma**	P-value*
Sex	Male	31.4	0.287	22.2	0.269	9.2	0.194
	Female	30.3		21.4		8.8	
Monthly income	1 (Lowest)	29.0	0.996	21.4	0.996	7.6	0.001
	2	30.6		21.7		8.9	
	3	30.2		21.6		8.6	
	4	28.4		21.7		6.7	
	5 (Highest)	37.4		22.7		14.8	
Age	<45 years	32.8	0.456	22.1	0.456	10.7	0.062
	45-64 years	29.5		21.7		7.9	
	≥65 years	28.1		20.6		7.5	
Residence	Rural	30.5	0.013	21.6	0.006	8.9	0.036
	Urban	39.3		25.6		13.7	
Education	Illiterate	30.6	0.531	21.6	0.531	9.1	0.794
	Up to primary schooling	31.8		22.6		8.6	
	Up to secondary schooling	30.2		21.6		9.0	
Comorbidities	Yes	33.2	0.554	22.1	0.965	10.9	0.350
	No	30.2		21.6		8.5	
Marital Status	Married	29.9	0.130	21.6	0.414	8.3	0.114
	Unmarried	31.8		21.9		9.9	
Disease Stage	Stage 1	25.2	0.005	19.7	0.002	5.5	0.001
	Stage 2	30.5		21.7		8.8	
	Stage 3	32.4		22.4		10.0	
	Stage 4	39.8		24.7		15.1	
	Stage 5	34.1		22.9		11.3	
Time lived in the current residence	≤5 years	36.5	0.804	23.1	0.804	13.1	0.359
	6-10 years	31.1		21.9		9.2	
	11-15 years	31.3		21.2		10.1	
	>15 years	30.4		21.7		8.7	

*Statistical significance was considered p < 0.05. Wilcoxon rank sum and Kruskal-Wallis tests were used as appropriate to derive the p-values.

A final adjusted linear regression model was controlled for age, education, marital status, place of residence, duration lived in current residence, monthly income, and disease stage as seen in Table 4. In this model, podoconiosis patients with a monthly income greater than the median were predicted to have a 5.18 point higher total mean stigma score (95% CI: 2.08 to 8.28) than those with a monthly income of less than the median (p = 0.001). Patients with disease stages 3 or higher were predicted to have 5.12 point higher total mean stigma score (95% CI: 2.06 to 8.18) than those with disease stage 1 (p = 0.001). Finally, as duration of residence in an area increased by one year, the stigma mean score decreased by 0.13 points (coeff = -0.13: 95% CI: -0.23 to -0.04) (p = 0.007).

**Table 4:** A linear regression analysis of total mean stigma scale scores and sociodemographic determinants among other factors in podoconiosis patients in Northern Ethiopia, May 2012 (N = 346)*

Variable*	**Unadjusted coefficient (95% CI)**	Adjusted coefficient (95% CI)**	**P-value**
Disease stage: Stage 3 or higher	4.73 (1.70 to 7.75)	5.12 (2.06 to 8.18)	0.001*
Monthly income: Greater than median	2.27 (-0.6 to 5.2)	5.18 (2.08 to 8.28)	0.001*
Marital status: Unmarried	1.92 (-1.0 to 4.8)	2.29 (-0.83 to 5.41)	0.149
Age in years 45-64	-2.38 (-5.39 to 0.64)	0.74 (-0.41 to 2.69)	0.671
Age in years > =65	-3.39 (-8.98 to 2.19)	0.58 (-5.67 to 6.83)	0.855
Education: Illiterate	2.56 (-0.87 to 5.98)	1.66 (-1.72 to 5.05)	0.335
Place of residence: Urban	8.75 (-1.43 to 18.93)	8.72 (-1.32 to 18.75)	0.088
Duration lived in the area £	-0.12 ( -0.19 to -0.03)	-0.13 (-0.23 to -0.04)	0.007*

*Reference groups: Disease stage (stage 1 and 2), Monthly income (less than median), Martial status (married), Age < 45, Education, literate, Place of residence, rural.

£ Duration lived in the area was treated as continuous variable.

**Adjusted coefficients were controlled for all other variables in the table.

## Discussion

This study chose to use the validated podoconiosis stigma scale consisting of distinct domains: felt and enacted stigma. As defined by Weiss *et al*. for NTDs, the hidden distress model distinguishes enacted stigma from felt stigma [28]. However, various measurements or definitions of stigma beyond the hidden distress model have been used in NTD-related stigma research, making direct study comparisons difficult [37]. This study aimed to first describe the stigma experience among podoconiosis patients by felt or enacted stigma and then to assess potential associations between stigma and sociodemographic determinants among other factors. There was a significantly higher mean stigma score of scale items in domains of felt stigma (highest in interpersonal interactions) as compared to enacted (highest in community, social and civic life). This is comparable to leprosy where felt stigma appears to be more widespread than enacted stigma [30].

Fewer analytical NTD-stigma studies were found than descriptive research. In this study, an association was found between stigma and factors including monthly income, marital status, and disease stage after controlling for confounders. Similarly, disease stage was found to be associated with stigma among LF patients in other studies [29,35]. This may be because increased podoconiosis disease stage is more visible than lower stages. Patients with advanced disease may not be able to hide from the community, exacerabating the extent of stigma felt or experienced. Young age was not found to be a significant risk factor in this study in contrast to other NTD-stigma studies [29,34]. Another study on leprosy chose to explore quality of life as the main outcome and found associations between decreased quality of life and stigma in addition to fewer years of education, the presence of deformities, and a lower annual income [37]. As shown in Figure 1, the mean stigma increases up to stage 4 steadily and decreases among stage 5. This might be due to the small sample of stage 5 patients rather than an actual trend.

Stigma research on TB and HIV/AIDS also provides comparisons. A study by Somma *et al*. explored determinants of TB-related stigma across Malawi, Bangladesh, and India. In Malawi, never having been married, reduced income and unemployment were associated with greater stigma. In India, never having been married and reduced income were assoiated with greater stigma. In Bangladesh, female sex, physical weakness and young age were associated with greater stigma [38]. For HIV/AIDS-related stigma, a study by Infante *et al*. identified gender, social class, and ethnicity as significiant determinants in Mexico whereas a study by Wong identified ethnicity, age, socioeconomic group, and an urban setting as significant determinants in Malaysia [39,40]. These results reflect region-specific differences.

In contrast to the TB and HIV/AIDS-related stigma research [5], this study found that individuals with higher income experienced or felt more stigma. This might be partly explained by patients with higher socioeconomic status being more socially and economically involved exposing them to further stigmatization than those who use avoidant coping strategies [12]. The difference in life experience between those with higher and lower socioeconomic status may significantly differ, resulting in differences in acceptable thresholds of stigma between these two groups. In further analysis the enacted stigma was significantly assoacited with dichotomized income (p = 0.003), whereas there was no assoacition between felt stigma and dichotomized income (p = 0.747). This needs further research to explore the interplay between income and stigma in this study setting. Finally, duration of stay in the current residence was found to predict stigma. As the duration increases, the level of stigma decreases, which might be due to the social networks established or more familiarity with individual patients.

Our finding suggests that felt stigma is more prevalent than enacted stigma. This might imply that patients tend to overestimate stigma. Efforts to diminish podoconiosis stigma should focus on counseling patients to live with podoconiosis and use a multidisciplinary approach to develop positive self-image through counseling.

This study had several strengths. The podoconiosis stigma scale tool used had proven consistency and validity in Ethiopia, although in a different community [17]. Nurses, with medical backgrounds, language fluency and understanding of local culture, acted as the data collectors. However, the study also had various limitations. Definitions of stigma often change across settings and time and can be difficult to measure, as summarized by Hotez *et al*. and reflected in several studies on TB and HIV/AIDS-related stigma [38-41]. Although validated, the scale may have some discrepancies across location or time. A mixed methods approach was not used in this study, so it lacked the qualitative depth displayed by other studies such as that by Stienstra *et al*. which used quantitative and qualitative data from interviews with patients and control subjects to study stigma towards Buruli ulcer [23]. Several groups were not studied to gain a full understanding of podoconiosis stigma, including community residents, health-care personnel, community leaders, and families of stigmatised individuals as identified by Weiss *et al*. [14]. The study design also did not allow for any comparisons of stigma across different settings such as the study by Person *et al*. which compared health-related stigma among women with lymphatic filariasis from the Dominican Republic and Ghana. Antecedents, consequences, coping strategies and outcomes indicated that Dominican women seemed far better off than Ghanaians in their experience of stigma [20]. Lastly, the use of nurses as data collectors in the community may have also biased the results if subjects felt pressurised to give answers or participate in the study (due to power differentials between the nurses and community members or misunderstandings about the difference between clinical versus research objectives). However, all research participants were informed about the study’s objectives and told that participation in the study would not affect their access to health services.

## Conclusion

Podoconiosis has a clear social impact. These social consequences and stigmatization are barriers to podoconiosis control and other research projects. Stigma related to podoconiosis needs to be addressed within the context of NTDs and global health. The link between global health and stigma has been largely recognized as evidenced by programmes such as the initiative launched by Fogarty International Center and partners and other leading research [42]. Weiss *et al*. proposed the following health-related stigma research priorities: (1) Document the burden of stigma, (2) Identify determinants of stigma and its effect, (3) Compare stigma for different health problems or settings, (4) Evaluate stigma over time or post-interventions, (5) Improve disease knowledge so that health policy minimises stigma, and (6) Develop appropriate messages about stigma [14]. This paper begins to address the first two objectives as they relate to podoconiosis, but further research is needed in view of the limitations of this study. Furthermore, comparative or evaluation studies should be conducted and evidence-based interventions to mitigate podoconiosis stigma should be pursued, particularly among patients with advanced disease stage.

## Competing interests

The authors declare that they have no competing interests.

## Authors’ contributions

KD, EM, GD conceived the study. EM, KD, AT conducted the field work. EM & KD conducted the analysis. KD, ST derafted the masnuscript. KD, ST, EM, AT and GD interpreted the findings. All authors read and approved the final manuscript.

## Pre-publication history

The pre-publication history for this paper can be accessed here:

http://www.biomedcentral.com/1471-2458/13/1178/prepub
